# Rate Distortion Function of Gaussian Asymptotically WSS Vector Processes

**DOI:** 10.3390/e20090719

**Published:** 2018-09-19

**Authors:** Jesús Gutiérrez-Gutiérrez, Marta Zárraga-Rodríguez, Pedro M. Crespo, Xabier Insausti

**Affiliations:** Tecnun, University of Navarra, Paseo de Manuel Lardizábal 13, 20018 San Sebastián, Spain

**Keywords:** rate distortion function, Gaussian vector processes, MA vector processes, ARMA vector processes, AWSS vector processes

## Abstract

In this paper, we obtain an integral formula for the rate distortion function (RDF) of any Gaussian asymptotically wide sense stationary (AWSS) vector process. Applying this result, we also obtain an integral formula for the RDF of Gaussian moving average (MA) vector processes and of Gaussian autoregressive MA (ARMA) AWSS vector processes.

## 1. Introduction

The present paper focuses on the derivation of a closed-form expression for the rate distortion function (RDF) of a wide class of vector processes. As stated in [1,2], there exist very few journal papers in the literature that present closed-form expressions for the RDF of non-stationary processes, and just one of them deals with non-stationary vector processes [3]. In the present paper, we obtain an integral formula for the RDF of any real Gaussian asymptotically wide sense stationary (AWSS) vector process. This new formula generalizes the one given in 1956 by Kolmogorov [4] for real Gaussian stationary processes and the one given in 1971 by Toms and Berger [3] for real Gaussian autoregressive (AR) AWSS vector processes of finite order. Applying this new formula, we also obtain an integral formula for the RDF of real Gaussian moving average (MA) vector processes of infinite order and for the RDF of real Gaussian ARMA AWSS vector processes of infinite order. AR, MA and ARMA vector processes are frequently used to model multivariate time series (see, e.g., [5]).

The definition of the AWSS process was first given by Gray (see [6,7]), and it is based on his concept of asymptotically equivalent sequences of matrices [8]. The integral formulas given in the present paper are obtained by using some recent results on such sequences of matrices [9,10,11,12].

The paper is organized as follows. In Section 2, we set up notation, and we review the concepts of AWSS, MA and ARMA vector processes and the Kolmogorov formula for the RDF of a real Gaussian vector. In Section 3, we obtain an integral formula for the RDF of any Gaussian AWSS vector process. In Section 4, we obtain an integral formula for the RDF of Gaussian MA vector processes and of Gaussian ARMA AWSS vector processes. We finish the paper with a numerical example where the RDF of a Gaussian AWSS vector process is computed.

## 2. Preliminaries

### 2.1. Notation

In this paper, N, Z, R and C denote the set of natural numbers (i.e., the set of positive integers), the set of integer numbers, the set of (finite) real numbers and the set of (finite) complex numbers, respectively. If m,n∈N, then $C^{m\times n}$, $0_{m\times n}$ and $I_{n}$ are the set of all m×n complex matrices, the m×n zero matrix and the n×n identity matrix, respectively. The symbols ⊤ and ∗ denote transpose and conjugate transpose, respectively. *E* stands for expectation; *i* is the imaginary unit; tr denotes trace; δ stands for the Kronecker delta; and $\lambda_{k}\left(A\right)$, k∈{1,…,n}, are the eigenvalues of an n×n Hermitian matrix *A* arranged in decreasing order.

Let $A_{n}$ and $B_{n}$ be nN×nN matrices for all n∈N. We write $\left\{A_{n}\right\}∼\left\{B_{n}\right\}$ if the sequences $\left\{A_{n}\right\}$ and $\left\{B_{n}\right\}$ are asymptotically equivalent (see ([9], p. 5673)), that is:

$$\exists M\in \left[0,\infty \right):\;∥A_{n}{∥}_{2},{∥B_{n}∥}_{2}\leq M\;\forall n\in N$$

and:

$$\lim_{n\to \infty }\frac{∥A_{n}-B_{n}{∥}_{F}}{\sqrt{n}}=0,$$

where ${∥\cdot ∥}_{2}$ and ${∥\cdot ∥}_{F}$ denote the spectral norm and the Frobenius norm, respectively. The original definition of asymptotically equivalent sequences of matrices, where N=1, was given by Gray (see ([6], Section 2.3) or [8]).

Let $\left\{x_{n}:n\in N\right\}$ be a random *N*-dimensional vector process, i.e., $x_{n}$ is a random (column) vector of dimension *N* for all n∈N. We denote by $x_{n:1}$ the random vector of dimension nN given by:

$$x_{n:1}:=\left(\begin{array}{c} x_{n}\\ x_{n-1}\\ x_{n-2}\\ \vdots \\ x_{1} \end{array}\right),\;n\in N.$$

Consider a matrix-valued function of a real variable $X:R\to C^{N\times N}$, which is continuous and 2π-periodic. For every n∈N, we denote by $T_{n}\left(X\right)$ the n×n block Toeplitz matrix with N×N blocks given by:

$$T_{n}\left(X\right):={\left(X_{j-k}\right)}_{j,k=1}^{n}=\left(\begin{array}{ccccc} X_{0} & X_{-1} & X_{-2} & \cdots & X_{1-n}\\ X_{1} & X_{0} & X_{-1} & \cdots & X_{2-n}\\ X_{2}\; & X_{1} & X_{0} & \cdots & X_{3-n}\\ \vdots & \vdots & \vdots & \ddots & \vdots \\ X_{n-1} & X_{n-2} & X_{n-3} & \cdots & X_{0} \end{array}\right),$$

where ${\left\{X_{k}\right\}}_{k\in Z}$ is the sequence of Fourier coefficients of *X*:

$$X_{k}=\frac{1}{2\pi }\int_{0}^{2\pi }e^{-k\omega i}X\left(\omega \right)d\omega \;\forall k\in Z.$$

### 2.2. AWSS Vector Processes

We first review the well-known concept of the WSS vector process.

**Definition** **1.** *Let $X:R\to C^{N\times N}$, and suppose that it is continuous and 2π-periodic. A random N-dimensional vector process $\left\{x_{n}:n\in N\right\}$ is said to be WSS (or weakly stationary) with power spectral density (PSD) X if it has constant mean (i.e., $E\left(x_{n_{1}}\right)=E\left(x_{n_{2}}\right)$ for all $n_{1},n_{2}\in N)$ and $\left\{E\left(x_{n:1}x_{n:1}^{*}\right)\right\}=\left\{T_{n}\left(X\right)\right\}$.*

We now review the definition of the AWSS vector process given in ([11], Definition 7.1).

**Definition** **2.** *Let $X:R\to C^{N\times N}$, and suppose that it is continuous and 2π-periodic. A random N-dimensional vector process $\left\{x_{n}:n\in N\right\}$ is said to be AWSS with asymptotic PSD (APSD) X if it has constant mean and $\left\{E\left(x_{n:1}x_{n:1}^{*}\right)\right\}∼\left\{T_{n}\left(X\right)\right\}$.*

Definition 2 was first introduced by Gray for the case N=1 (see, e.g., ([6], p. 225)).

### 2.3. MA and ARMA Vector Processes

We first review the concept of real zero-mean MA vector process (of infinite order).

**Definition** **3.** *A real zero-mean random N-dimensional vector process $\left\{x_{n}:n\in N\right\}$ is said to be MA if:*

(1) $$x_{n}=w_{n}+\sum_{j=1}^{n-1}G_{-j}w_{n-j}\;\forall n\in N,$$

*where $G_{-j}$, j∈N, are real N×N matrices, $\left\{w_{n}:n\in N\right\}$ is a real zero-mean random N-dimensional vector process and $E\left(w_{n_{1}}w_{n_{2}}^{⊤}\right)=\delta_{n_{1},n_{2}}\Lambda$ for all $n_{1},n_{2}\in N$ with *Λ* being an N×N positive definite matrix.*

The MA vector process $\left\{x_{n}:n\in N\right\}$ in Equation (1) is of finite order if there exists q∈N such that $G_{-j}=0_{N\times N}$ for all j>q. In this case, $\left\{x_{n}:n\in N\right\}$ is called an MA(q) vector process (see, e.g., ([5], Section 2.1)).

Secondly, we review the concept of a real zero-mean ARMA vector process (of infinite order).

**Definition** **4.** *A real zero-mean random N-dimensional vector process $\left\{x_{n}:n\in N\right\}$ is said to be ARMA if:*

(2) $$x_{n}=w_{n}+\sum_{j=1}^{n-1}G_{-j}w_{n-j}-\sum_{j=1}^{n-1}F_{-j}x_{n-j}\;\forall n\in N,$$

*where $G_{-j}$ and $F_{-j}$, j∈N, are real N×N matrices, $\left\{w_{n}:n\in N\right\}$ is a real zero-mean random N-dimensional vector process and $E\left(w_{n_{1}}w_{n_{2}}^{⊤}\right)=\delta_{n_{1},n_{2}}\Lambda$ for all $n_{1},n_{2}\in N$ with *Λ* being an N×N positive definite matrix.*

The ARMA vector process $\left\{x_{n}:n\in N\right\}$ in Equation (2) is of finite order if there exist p,q∈N such that $F_{-j}=0_{N\times N}$ for all j>p and $G_{-j}=0_{N\times N}$ for all j>q. In this case, $\left\{x_{n}:n\in N\right\}$ is called an ARMA(p,q) vector process (see, e.g., ([5], Section 1.2.2)).

### 2.4. RDF of Gaussian Vectors

Let $\left\{x_{n}:n\in N\right\}$ be a real zero-mean Gaussian *N*-dimensional vector process satisfying that $E\left(x_{n:1}x_{n:1}^{⊤}\right)$ is positive definite for all n∈N. If n∈N from [4], we know that the RDF of the real zero-mean Gaussian vector $x_{n:1}$ is given by:

(3) $$R_{n}\left(D\right)=\frac{1}{nN}\sum_{k=1}^{nN}\max\left\{0,\frac{1}{2}\ln\frac{\lambda_{k}\left(E\left(x_{n:1}x_{n:1}^{⊤}\right)\right)}{\theta_{n}}\right\}$$

with $D\in \left(0,\frac{\mathrm{tr}\left(E\left(x_{n:1}x_{n:1}^{⊤}\right)\right)}{nN}\right)$ and where $\theta_{n}$ is the real number satisfying:

$$D=\frac{1}{nN}\sum_{k=1}^{nN}\min\left\{\theta_{n},\lambda_{k}\left(E\left(x_{n:1}x_{n:1}^{⊤}\right)\right)\right\}.$$

The RDF of the real zero-mean Gaussian vector process $\left\{x_{n}:n\in N\right\}$ is given by:

$$R\left(D\right):=\lim_{n\to \infty }R_{n}\left(D\right)$$

whenever this limit exists.

## 3. Integral Formula for the RDF of Gaussian AWSS Vector Processes

**Theorem** **1.** *Let $\left\{x_{n}:n\in N\right\}$ be a real zero-mean Gaussian AWSS N-dimensional vector process with APSD X. Suppose that X(ω) is positive definite for all ω∈R and that $E\left(x_{n:1}x_{n:1}^{⊤}\right)$ is positive definite for all n∈N. If $D\in \left(0,\frac{\mathrm{tr}(X_{0})}{N}\right)$, then:*

(4) $$R\left(D\right)=\frac{1}{4\pi N}\int_{0}^{2\pi }\sum_{k=1}^{N}\max\left\{0,\ln\frac{\lambda_{k}\left(X(\omega )\right)}{\theta }\right\}d\omega$$

*is the operational RDF of $\left\{x_{n}:n\in N\right\}$, where θ is the real number satisfying:*

(5) $$D=\frac{1}{2\pi N}\int_{0}^{2\pi }\sum_{k=1}^{N}\min\left\{\theta ,\lambda_{k}\left(X(\omega )\right)\right\}d\omega .$$

**Proof.**  See Appendix A. ☐

**Corollary** **1.** *Let $\left\{x_{n}:n\in N\right\}$ be a real zero-mean Gaussian WSS N-dimensional vector process with PSD X. Suppose that X(ω) is positive definite for all ω∈R. If $D\in \left(0,\frac{\mathrm{tr}(X_{0})}{N}\right)$, then:*

(6) $$R\left(D\right)=\frac{1}{4\pi N}\int_{0}^{2\pi }\sum_{k=1}^{N}\max\left\{0,\ln\frac{\lambda_{k}\left(X(\omega )\right)}{\theta }\right\}d\omega ,$$

*where θ is the real number satisfying:*

$$D=\frac{1}{2\pi N}\int_{0}^{2\pi }\sum_{k=1}^{N}\min\left\{\theta ,\lambda_{k}\left(X(\omega )\right)\right\}d\omega .$$

**Proof.**  See Appendix B. ☐

The integral formula given in Equation (6) was presented by Kafedziski in ([13], Equation (20)). However, the proof that he proposed was not complete, because although Kafedziski pointed out that ([13], Equation (20)) can be directly proven by applying the Szegö theorem for block Toeplitz matrices ([14], Theorem 3), the Szegö theorem cannot be applied since the parameter θ that appears in the expression of $R_{n}\left(D\right)$ in ([13], Equation (7)), depends on *n*, as it does in Equation (3). It should be also mentioned that the set of WSS vector processes that he considered was smaller, namely, he only considered WSS vector processes with PSD in the Wiener class. A function $X:R\to C^{N\times N}$ is said to be in the Wiener class if it is continuous and 2π-periodic, and it satisfies $\sum_{k=-\infty }^{\infty }\left|{\left[X_{k}\right]}_{r,s}\right|<\infty$ for all r,s∈{1,…,N} (see, e.g., ([11], Appendix B)).

## 4. Applications

### 4.1. Integral Formula for the RDF of Gaussian MA Vector Processes

**Theorem** **2.** *Let $\left\{x_{n}:n\in N\right\}$ be as in Definition 3. Assume that ${\left\{G_{k}\right\}}_{k=-\infty }^{\infty }$, with $G_{0}=I_{N}$ and $G_{k}=0_{N\times N}$ for all k>0, is the sequence of Fourier coefficients of a function $G:R\to C^{N\times N}$, which is continuous and 2π-periodic. Then:*

*1.*  *$\left\{x_{n}:n\in N\right\}$ is AWSS with APSD $X\left(\omega \right)=G\left(\omega \right)\Lambda {\left(G\left(\omega \right)\right)}^{*}$ for all ω∈R.*
*2.*  *If $\left\{x_{n}:n\in N\right\}$ is Gaussian, det(G(ω))≠0 for all ω∈R, and $D\in \left(0,\frac{\mathrm{tr}(X_{0})}{N}\right)$ yields*
  $$R\left(D\right)=\frac{1}{4\pi N}\int_{0}^{2\pi }\sum_{k=1}^{N}\max\left\{0,\ln\frac{\lambda_{k}\left(G\left(\omega \right)\Lambda {\left(G\left(\omega \right)\right)}^{*}\right)}{\theta }\right\}d\omega ,$$
  *where θ is the real number satisfying:*
  $$D=\frac{1}{2\pi N}\int_{0}^{2\pi }\sum_{k=1}^{N}\min\left\{\theta ,\lambda_{k}\left(G\left(\omega \right)\Lambda {\left(G\left(\omega \right)\right)}^{*}\right)\right\}d\omega .$$

**Proof.**  See Appendix C. ☐

### 4.2. Integral Formula for the RDF of Gaussian ARMA AWSS Vector Processes

**Theorem** **3.** *Let $\left\{x_{n}:n\in N\right\}$ be as in Definition 4. Assume that ${\left\{G_{k}\right\}}_{k=-\infty }^{\infty }$, with $G_{0}=I_{N}$ and $G_{k}=0_{N\times N}$ for all k>0, is the sequence of Fourier coefficients of a function $G:R\to C^{N\times N}$, which is continuous and 2π-periodic. Suppose that ${\left\{F_{k}\right\}}_{k=-\infty }^{\infty }$, with $F_{0}=I_{N}$ and $F_{k}=0_{N\times N}$ for all k>0, is the sequence of Fourier coefficients of a function $F:R\to C^{N\times N}$, which is continuous and 2π-periodic. Assume that $\left\{∥{\left(T_{n}\left(F\right)\right)}^{-1}{∥}_{2}\right\}$ is bounded and det(F(ω))≠0 for all ω∈R. Then:*

*1.*  *$\left\{x_{n}:n\in N\right\}$ is AWSS with APSD $X\left(\omega \right)={\left(F\left(\omega \right)\right)}^{-1}G\left(\omega \right)\Lambda {\left({\left(F\left(\omega \right)\right)}^{-1}G\left(\omega \right)\right)}^{*}$ for all ω∈R.*
*2.*  *If $\left\{x_{n}:n\in N\right\}$ is Gaussian, det(G(ω))≠0 for all ω∈R, and $D\in \left(0,\frac{\mathrm{tr}(X_{0})}{N}\right)$ yields:*
  $$R\left(D\right)=\frac{1}{4\pi N}\int_{0}^{2\pi }\sum_{k=1}^{N}\max\left\{0,\ln\frac{\lambda_{k}\left({\left(F\left(\omega \right)\right)}^{-1}G\left(\omega \right)\Lambda {\left({\left(F\left(\omega \right)\right)}^{-1}G\left(\omega \right)\right)}^{*}\right)}{\theta }\right\}d\omega ,$$
  *where θ is the real number satisfying:*
  $$D=\frac{1}{2\pi N}\int_{0}^{2\pi }\sum_{k=1}^{N}\min\left\{\theta ,\lambda_{k}\left({\left(F\left(\omega \right)\right)}^{-1}G\left(\omega \right)\Lambda {\left({\left(F\left(\omega \right)\right)}^{-1}G\left(\omega \right)\right)}^{*}\right)\right\}d\omega .$$

**Proof.**  See Appendix D. ☐

## 5. Numerical Example

We finish the paper with a numerical example where the RDF of a Gaussian AWSS vector process is computed. Specifically, we compute the RDF of the MA(1) vector process considered in ([5], Example 2.1), by assuming that it is Gaussian.

Let $\left\{x_{n}:n\in N\right\}$ be as in Definition 3 with N=2,

$$G_{-1}=\left(\begin{array}{cc} -0.8 & -0.7\\ 0.4 & -0.6 \end{array}\right),$$

$G_{-j}=0_{2\times 2}$ for all j>1, and:

$$\Lambda =\left(\begin{array}{cc} 4 & 1\\ 1 & 2 \end{array}\right).$$

**Figure 1:**
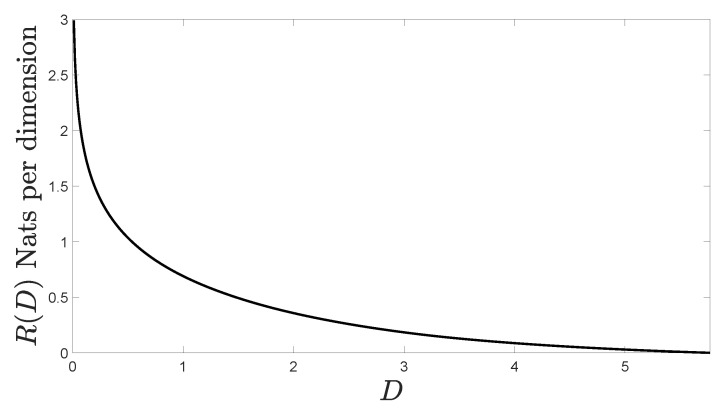
Rate Distortion Function (RDF) of the Gaussian MA vector process considered.

Assume that $\left\{x_{n}:n\in N\right\}$ is Gaussian. Figure 1 shows R(D) with D∈(0,5.77) that we have computed using Theorem 2.

## Appendix A. Proof of Theorem 1

**Proof.** We divide the proof into six steps. Step 1: We show that there exists $n_{0}\in N$ such that $\theta_{n}$ in Equation (3) exists for all $n\geq n_{0}$, or equivalently, such that $D\in \left(0,\frac{\mathrm{tr}\left(E\left(x_{n:1}x_{n:1}^{⊤}\right)\right)}{nN}\right)$ for all $n\geq n_{0}$. Since $\left\{E\left(x_{n:1}x_{n:1}^{⊤}\right)\right\}=\left\{E\left(x_{n:1}x_{n:1}^{*}\right)\right\}∼\left\{T_{n}\left(X\right)\right\}$, applying ([11], Theorem 6.6) yields:

(A1) $$\begin{array}{cc} \lim_{n\to \infty }\frac{1}{nN}\sum_{k=1}^{nN}\lambda_{k}\left(E\left(x_{n:1}x_{n:1}^{⊤}\right)\right) & =\frac{1}{2\pi N}\int_{0}^{2\pi }\sum_{k=1}^{N}\lambda_{k}\left(X(\omega )\right)d\omega =\frac{1}{2\pi N}\int_{0}^{2\pi }\mathrm{tr}\left(X(\omega )\right)d\omega \\ & =\frac{1}{2\pi N}\int_{0}^{2\pi }\sum_{k=1}^{N}{\left[X\left(\omega \right)\right]}_{k,k}d\omega =\frac{1}{2\pi N}\sum_{k=1}^{N}\int_{0}^{2\pi }{\left[X\left(\omega \right)\right]}_{k,k}d\omega \\ & =\frac{1}{2\pi N}\sum_{k=1}^{N}{\left[\int_{0}^{2\pi }X\left(\omega \right)d\omega \right]}_{k,k}=\frac{1}{2\pi N}\mathrm{tr}\left(\int_{0}^{2\pi }X\left(\omega \right)d\omega \right)\\ & =\frac{1}{N}\mathrm{tr}\left(\frac{1}{2\pi }\int_{0}^{2\pi }X\left(\omega \right)d\omega \right)=\frac{\mathrm{tr}(X_{0})}{N}. \end{array}$$

Consequently, as $D\in \left(0,\frac{\mathrm{tr}(X_{0})}{N}\right)$, there exists $n_{0}\in N$ such that:

$$\left|\frac{1}{nN}\sum_{k=1}^{nN}\lambda_{k}\left(E\left(x_{n:1}x_{n:1}^{⊤}\right)\right)-\frac{\mathrm{tr}(X_{0})}{N}\right|<\frac{\mathrm{tr}(X_{0})}{N}-D\;\forall n\geq n_{0}.$$

Therefore, since:

$$\frac{\mathrm{tr}(X_{0})}{N}-\frac{1}{nN}\sum_{k=1}^{nN}\lambda_{k}\left(E\left(x_{n:1}x_{n:1}^{⊤}\right)\right)\leq \left|\frac{1}{nN}\sum_{k=1}^{nN}\lambda_{k}\left(E\left(x_{n:1}x_{n:1}^{⊤}\right)\right)-\frac{\mathrm{tr}(X_{0})}{N}\right|\;\forall n\geq n_{0},$$

we obtain:

(A2) $$D<\frac{1}{nN}\sum_{k=1}^{nN}\lambda_{k}\left(E\left(x_{n:1}x_{n:1}^{⊤}\right)\right)=\frac{\mathrm{tr}\left(E\left(x_{n:1}x_{n:1}^{⊤}\right)\right)}{nN}\;\forall n\geq n_{0}.$$

Step 2: We prove that the sequence of real numbers ${\left\{\theta_{n}\right\}}_{n\geq n_{0}}$ is bounded. From Equation (A2), we have $\theta_{n}<\lambda_{1}\left(E\left(x_{n:1}x_{n:1}^{⊤}\right)\right)$ for all $n\geq n_{0}$. As $\left\{E\left(x_{n:1}x_{n:1}^{⊤}\right)\right\}∼\left\{T_{n}\left(X\right)\right\}$, there exists M∈[0,∞) such that $∥E\left(x_{n:1}x_{n:1}^{⊤}\right){∥}_{2},{∥T_{n}\left(X\right)∥}_{2}\leq M$ for all n∈N. Thus,

$$\begin{array}{c} 0\;<\;D=\frac{1}{nN}\sum_{k=1}^{nN}\min\left\{\theta_{n},\lambda_{k}\left(E\left(x_{n:1}x_{n:1}^{⊤}\right)\right)\right\}\leq \frac{1}{nN}\sum_{k=1}^{nN}\theta_{n}\\ =\theta_{n}<\lambda_{1}\left(E\left(x_{n:1}x_{n:1}^{⊤}\right)\right)\;=\;{∥E\left(x_{n:1}x_{n:1}^{⊤}\right)∥}_{2}\leq M\;\forall n\geq n_{0}. \end{array}$$

Step 3: We show that if $\left\{\theta_{\sigma (n)}\right\}$ is a convergent subsequence of ${\left\{\theta_{n}\right\}}_{n\geq n_{0}}$, then $\lim_{n\to \infty }\theta_{\sigma (n)}=\theta$. We denote by $\hat{\theta }$ the limit of $\left\{\theta_{\sigma (n)}\right\}$. We need to prove that $\hat{\theta }=\theta$. Since $0<D\leq \theta_{n}$ for all $n\geq n_{0}$, we have $0<D\leq \hat{\theta }$. Let $\left\{{\hat{\theta }}_{n}\right\}$ be the sequence of real numbers such that $\left\{{\hat{\theta }}_{\sigma (n)}\right\}=\left\{\theta_{\sigma (n)}\right\}$ and ${\hat{\theta }}_{n}=\hat{\theta }$ for all n∈N\σ(N). Obviously, $\lim_{n\to \infty }{\hat{\theta }}_{n}=\hat{\theta }$ and $0<{\hat{\theta }}_{n}$ for all n∈N. As $\lim_{n\to \infty }\frac{1}{{\hat{\theta }}_{n}}=\frac{1}{\hat{\theta }}$ and $\frac{\mathrm{rank}\left(E\left(x_{n:1}x_{n:1}^{⊤}\right)\right)}{n}=N$ for all n∈N, applying ([12], Lemma 1) yields $\left\{\frac{1}{{\hat{\theta }}_{n}}E\left(x_{n:1}x_{n:1}^{⊤}\right)\right\}∼\left\{\frac{1}{\hat{\theta }}T_{n}\left(X\right)\right\}$. From ([11], Lemma 4.2) we obtain $\left\{\frac{1}{{\hat{\theta }}_{n}}E\left(x_{n:1}x_{n:1}^{⊤}\right)\right\}∼\left\{\frac{1}{\hat{\theta }}T_{n}\left(X\right)\right\}=\left\{T_{n}\left(\frac{1}{\hat{\theta }}X\right)\right\}$. Hence, applying ([11], Theorem 6.6) yields:

$$\begin{array}{cc} D & =\lim_{n\to \infty }\frac{1}{\sigma (n)N}\sum_{k=1}^{\sigma (n)N}\min\left\{\theta_{\sigma (n)},\lambda_{k}\left(E\left(x_{\sigma (n):1}x_{\sigma (n):1}^{⊤}\right)\right)\right\}\\ & =\lim_{n\to \infty }\theta_{\sigma (n)}\frac{1}{\sigma (n)N}\sum_{k=1}^{\sigma (n)N}\min\left\{1,\frac{\lambda_{k}\left(E\left(x_{\sigma (n):1}x_{\sigma (n):1}^{⊤}\right)\right)}{\theta_{\sigma (n)}}\right\}\\ & =\hat{\theta }\lim_{n\to \infty }\frac{1}{\sigma (n)N}\sum_{k=1}^{\sigma (n)N}\min\left\{1,\frac{\lambda_{k}\left(E\left(x_{\sigma (n):1}x_{\sigma (n):1}^{⊤}\right)\right)}{{\hat{\theta }}_{\sigma (n)}}\right\}\\ & =\hat{\theta }\lim_{n\to \infty }\frac{1}{nN}\sum_{k=1}^{nN}\min\left\{1,\frac{\lambda_{k}\left(E\left(x_{n:1}x_{n:1}^{⊤}\right)\right)}{{\hat{\theta }}_{n}}\right\}\\ & =\hat{\theta }\lim_{n\to \infty }\frac{1}{nN}\sum_{k=1}^{nN}\min\left\{1,\lambda_{k}\left(\frac{1}{{\hat{\theta }}_{n}}E\left(x_{n:1}x_{n:1}^{⊤}\right)\right)\right\}\\ & =\hat{\theta }\frac{1}{2\pi N}\int_{0}^{2\pi }\sum_{k=1}^{N}\min\left\{1,\lambda_{k}\left(\frac{1}{\hat{\theta }}X\left(\omega \right)\right)\right\}d\omega \\ & =\hat{\theta }\frac{1}{2\pi N}\int_{0}^{2\pi }\sum_{k=1}^{N}\min\left\{1,\frac{\lambda_{k}\left(X(\omega )\right)}{\hat{\theta }}\right\}d\omega \\ & =\frac{1}{2\pi N}\int_{0}^{2\pi }\sum_{k=1}^{N}\min\left\{\hat{\theta },\lambda_{k}\left(X(\omega )\right)\right\}d\omega . \end{array}$$

Thus, $\hat{\theta }$ is a real number satisfying Equation (5). Since $D<\frac{\mathrm{tr}(X_{0})}{N}=\frac{1}{2\pi N}\int_{0}^{2\pi }\sum_{k=1}^{N}\lambda_{k}\left(X(\omega )\right)d\omega$, there exists a unique real number θ satisfying Equation (5), and consequently, $\hat{\theta }=\theta$. Step 4: We prove that $\lim_{n\to \infty }\theta_{n}=\theta$. From Steps 2 and 3, we have ${\lim\;\inf}_{n\to \infty }\theta_{n}={\lim\;\sup}_{n\to \infty }\theta_{n}=\theta$. Consequently, the sequence of real numbers ${\left\{\theta_{n}\right\}}_{n\geq n_{0}}$ is convergent, and its limit is θ (see, e.g., ([15], p. 57)). Step 5: We show that Equation (4) holds. Let $\left\{{\hat{\theta }}_{n}\right\}$ be the sequence of positive numbers defined in Step 3 for the case in which $\left\{\sigma \left(n\right)\right\}=\left\{n+n_{0}-1\right\}$, that is, ${\hat{\theta }}_{n}=\theta_{n}$ if $n\geq n_{0}$ and ${\hat{\theta }}_{n}=\theta$ if $n<n_{0}$. From ([11], Theorem 6.6), we obtain:

$$\begin{array}{cc} R(D) & =\lim_{n\to \infty }R_{n}\left(D\right)=\lim_{n\to \infty }\frac{1}{nN}\sum_{k=1}^{nN}\max\left\{0,\frac{1}{2}\ln\frac{\lambda_{k}\left(E\left(x_{n:1}x_{n:1}^{⊤}\right)\right)}{\theta_{n}}\right\}\\ & =\lim_{n\to \infty }\frac{1}{nN}\sum_{k=1}^{nN}\frac{1}{2}\ln\max\left\{1,\frac{\lambda_{k}\left(E\left(x_{n:1}x_{n:1}^{⊤}\right)\right)}{\theta_{n}}\right\}=\frac{1}{2}\lim_{n\to \infty }\frac{1}{nN}\sum_{k=1}^{nN}\ln\max\left\{1,\frac{\lambda_{k}\left(E\left(x_{n:1}x_{n:1}^{⊤}\right)\right)}{{\hat{\theta }}_{n}}\right\}\\ & =\frac{1}{2}\lim_{n\to \infty }\frac{1}{nN}\sum_{k=1}^{nN}\ln\max\left\{1,\lambda_{k}\left(\frac{1}{{\hat{\theta }}_{n}}E\left(x_{n:1}x_{n:1}^{⊤}\right)\right)\;\;\right\}\;=\;\frac{1}{4\pi N}\int_{0}^{2\pi }\;\sum_{k=1}^{N}\ln\max\left\{1,\lambda_{k}\left(\frac{1}{\theta }X\left(\omega \right)\right)\right\}d\omega \\ & =\frac{1}{4\pi N}\;\int_{0}^{2\pi }\;\sum_{k=1}^{N}\ln\max\left\{1,\frac{\lambda_{k}\left(X(\omega )\right)}{\theta }\right\}\;d\omega =\frac{1}{4\pi N}\int_{0}^{2\pi }\sum_{k=1}^{N}\max\left\{0,\ln\frac{\lambda_{k}\left(X(\omega )\right)}{\theta }\right\}d\omega . \end{array}$$

Step 6: We prove that Equation (4) is the operational RDF of $\left\{x_{n}:n\in N\right\}$. Following the same arguments that Gray used in [16] for Gaussian AR AWSS one-dimensional vector processes, to prove the negative (converse) coding theorem and the positive (achievability) coding theorem, we only need to show that the sequence $\left\{d_{\max}^{*}\left(n\right)\right\}$ defined in ([17], p. 490), is bounded. Hence, Equation (A1) finishes the proof. ☐

## Appendix B. Proof of Corollary 1

**Proof.** Since X(ω) is positive definite for all ω∈R, from ([11], Theorem 4.4) and ([18], Corollary VI.1.6), we have:

$$0\;<\;\min_{\omega \in [0,2\pi ]}\lambda_{N}\left(X(\omega )\right)\;=\;\underset{\omega \in [0,2\pi ]}{\inf}\lambda_{N}\left(X(\omega )\right)\;\leq \;\lambda_{nN}\left(T_{n}\left(X\right)\right)\;=\;\lambda_{nN}\left(E\left(x_{n:1}x_{n:1}^{*}\right)\right)\;=\;\lambda_{nN}\left(E\left(x_{n:1}x_{n:1}^{⊤}\right)\right)$$

for all n∈N, and consequently, $E\left(x_{n:1}x_{n:1}^{⊤}\right)$ is positive definite for all n∈N. Combining ([11], Lemma 3.3) and ([11], Theorem 4.3) yields $\left\{E\left(x_{n:1}x_{n:1}^{*}\right)\right\}=\left\{T_{n}\left(X\right)\right\}∼\left\{T_{n}\left(X\right)\right\}$. The proof finishes by applying Theorem 1. ☐

## Appendix C. Proof of Theorem 2

**Proof.** (1) From Equation (1), we have:

$$\left(\begin{array}{c} x_{n}\\ x_{n-1}\\ x_{n-2}\\ \vdots \\ x_{1} \end{array}\right)=\left(\begin{array}{ccccc} I_{N} & G_{-1} & G_{-2} & \cdots & G_{1-n}\\ 0_{N\times N} & I_{N} & G_{-1} & \cdots & G_{2-n}\\ 0_{N\times N}\; & 0_{N\times N} & I_{N} & \cdots & G_{3-n}\\ \vdots & \vdots & \vdots & \ddots & \vdots \\ 0_{N\times N} & 0_{N\times N} & 0_{N\times N} & \cdots & I_{N} \end{array}\right)\left(\begin{array}{c} w_{n}\\ w_{n-1}\\ w_{n-2}\\ \vdots \\ w_{1} \end{array}\right),$$

or more compactly,

$$x_{n:1}=T_{n}\left(G\right)w_{n:1}$$

for all n∈N. Consequently,

$$x_{n:1}x_{n:1}^{⊤}=T_{n}\left(G\right)w_{n:1}w_{n:1}^{⊤}{\left(T_{n}\left(G\right)\right)}^{⊤}\;\forall n\in N,$$

and applying ([11], Lemma 4.2), yields:

(A3) $$\begin{array}{cc} \left\{E\left(x_{n:1}x_{n:1}^{⊤}\right)\right\} & =\left\{T_{n}\left(G\right)E\left(w_{n:1}w_{n:1}^{⊤}\right){\left(T_{n}\left(G\right)\right)}^{⊤}\right\}\\ & =\left\{T_{n}\left(G\right)T_{n}\left(\Lambda \right){\left(T_{n}\left(G\right)\right)}^{*}\right\}=\left\{T_{n}\left(G\right)T_{n}\left(\Lambda \right)T_{n}\left(G^{*}\right)\right\}, \end{array}$$

where $G^{*}\left(\omega \right)={\left(G\left(\omega \right)\right)}^{*}$, ω∈R. Combining ([11], Lemma 3.3) and ([11], Theorem 4.3), we obtain $\left\{T_{n}\left(G\right)\right\}∼\left\{T_{n}\left(G\right)\right\}$. Moreover, applying ([10], Theorem 3) yields $\left\{T_{n}\left(\Lambda \right)T_{n}\left(G^{*}\right)\right\}∼\left\{T_{n}\left(\Lambda G^{*}\right)\right\}$. Hence, from ([10], Lemma 2) and ([10], Theorem 3), we have:

(A4) $$\begin{array}{cc} \left\{E\left(x_{n:1}x_{n:1}^{*}\right)\right\} & =\left\{E\left(x_{n:1}x_{n:1}^{⊤}\right)\right\}=\left\{T_{n}\left(G\right)T_{n}\left(\Lambda \right)T_{n}\left(G^{*}\right)\right\}\\ & ∼\left\{T_{n}\left(G\right)T_{n}\left(\Lambda G^{*}\right)\right\}∼\left\{T_{n}\left(G\Lambda G^{*}\right)\right\}=\left\{T_{n}\left(X\right)\right\}. \end{array}$$

Thus, as the relation ∼ is transitive (see ([11], Lemma 3.1)), $\left\{x_{n}:n\in N\right\}$ is AWSS with APSD *X*. (2) First, we prove that X(ω) is positive definite for all ω∈R. Fix ω∈R, and consider $y\in C^{N\times 1}$. Since Λ is positive definite, we have:

$$y^{*}X\left(\omega \right)y=y^{*}G\left(\omega \right)\Lambda {\left(G\left(\omega \right)\right)}^{*}y={\left({\left(G\left(\omega \right)\right)}^{*}y\right)}^{*}\Lambda {\left(G\left(\omega \right)\right)}^{*}y>0$$

whenever ${\left(G\left(\omega \right)\right)}^{*}y\neq 0_{N\times 1}$. As det(G(ω))≠0, ${\left(G\left(\omega \right)\right)}^{*}y=0_{N\times 1}$ if and only if $y={\left({\left(G\left(\omega \right)\right)}^{-1}\right)}^{*}0_{N\times 1}=0_{N\times 1}$, and consequently, X(ω) is positive definite. Secondly, we prove that $E\left(x_{n:1}x_{n:1}^{⊤}\right)$ is positive definite for all n∈N. To do that, we only need to show that $\det\left(E\left(x_{n:1}x_{n:1}^{⊤}\right)\right)\neq 0$ for all n∈N, because as $E\left(x_{n:1}x_{n:1}^{⊤}\right)$ is a correlation matrix, it is positive semidefinite. We have:

(A5) $$\begin{array}{cc} \det\left(E\left(x_{n:1}x_{n:1}^{⊤}\right)\right) & \;=\;\det\left(T_{n}\left(G\right)T_{n}\left(\Lambda \right)T_{n}\left(G^{*}\right)\right)\;=\;\det\left(T_{n}\left(G\right)\right)\det\left(T_{n}\left(\Lambda \right)\right)\det\left({\left(T_{n}\left(G\right)\right)}^{*}\right)\\ & \;=\;|\det\left(T_{n}\left(G\right)\right){|}^{2}{\left(\det(\Lambda )\right)}^{n}\;=\;{\left|{\left(\det(I_{N})\right)}^{n}\right|}^{2}{\left(\det(\Lambda )\right)}^{n}\;=\;{\left(\det(\Lambda )\right)}^{n}\neq 0 \end{array}$$

for all n∈N. The result now follows from Theorem 1. ☐

## Appendix D. Proof of Theorem 3

**Proof.** (1) From Equation (2), we have:

$$\sum_{j=0}^{n-1}F_{-j}x_{n-j}=\sum_{j=0}^{n-1}G_{-j}w_{n-j},$$

or more compactly,

$$T_{n}\left(F\right)x_{n:1}=T_{n}\left(G\right)w_{n:1}$$

for all n∈N. Consequently,

$$T_{n}\left(F\right)x_{n:1}x_{n:1}^{⊤}{\left(T_{n}\left(F\right)\right)}^{⊤}=T_{n}\left(F\right)x_{n:1}{\left(T_{n}\left(F\right)x_{n:1}\right)}^{⊤}=T_{n}\left(G\right)w_{n:1}w_{n:1}^{⊤}{\left(T_{n}\left(G\right)\right)}^{⊤}\;\forall n\in N,$$

and applying Equation (A3) yields:

$$\begin{array}{cc} \left\{T_{n}\left(F\right)E\left(x_{n:1}x_{n:1}^{*}\right){\left(T_{n}\left(F\right)\right)}^{*}\right\} & =\left\{T_{n}\left(F\right)E\left(x_{n:1}x_{n:1}^{⊤}\right){\left(T_{n}\left(F\right)\right)}^{⊤}\right\}\\ & =\left\{T_{n}\left(G\right)E\left(w_{n:1}w_{n:1}^{⊤}\right){\left(T_{n}\left(G\right)\right)}^{⊤}\right\}=\left\{T_{n}\left(G\right)T_{n}\left(\Lambda \right)T_{n}\left(G^{*}\right)\right\}. \end{array}$$

Since $\det\left(T_{n}\left(F\right)\right)={\left(\det\left(I_{N}\right)\right)}^{n}=1\neq 0$ for all n∈N, we obtain:

$$\begin{array}{cc} \left\{E\left(x_{n:1}x_{n:1}^{*}\right)\right\} & =\left\{{\left(T_{n}\left(F\right)\right)}^{-1}T_{n}\left(G\right)T_{n}\left(\Lambda \right)T_{n}\left(G^{*}\right){\left({\left(T_{n}\left(F\right)\right)}^{*}\right)}^{-1}\right\}\\ & =\left\{{\left({\left(T_{n}\left(F\right)\right)}^{*}T_{n}\left(F\right)\right)}^{-1}{\left(T_{n}\left(F\right)\right)}^{*}T_{n}\left(G\right)T_{n}\left(\Lambda \right)T_{n}\left(G^{*}\right)T_{n}\left(F\right){\left({\left(T_{n}\left(F\right)\right)}^{*}T_{n}\left(F\right)\right)}^{-1}\right\}. \end{array}$$

From Equation (A4) and the fact that the relation ∼ is transitive (see ([11], Lemma 3.1)), we have $\left\{T_{n}\left(G\right)T_{n}\left(\Lambda \right)T_{n}\left(G^{*}\right)\right\}∼\left\{T_{n}\left(G\Lambda G^{*}\right)\right\}$. Combining ([11], Lemma 3.3) and ([11], Theorem 4.3) yields $\left\{T_{n}\left(F\right)\right\}∼\left\{T_{n}\left(F\right)\right\}$. Therefore, applying ([10], Lemma 2) and ([10], Theorem 3), we obtain:

$$\left\{T_{n}\left(G\right)T_{n}\left(\Lambda \right)T_{n}\left(G^{*}\right)T_{n}\left(F\right)\right\}∼\left\{T_{n}\left(G\Lambda G^{*}\right)T_{n}\left(F\right)\right\}∼\left\{T_{n}\left(G\Lambda G^{*}F\right)\right\}.$$

Using ([11], Lemma 3.1) yields $\left\{{\left(T_{n}\left(F\right)\right)}^{*}\right\}∼\left\{{\left(T_{n}\left(F\right)\right)}^{*}\right\}$, and applying ([10], Lemma 2), ([11], Lemma 4.2), and ([10], Theorem 3), we have:

(A6) $$\begin{array}{cc} \left\{{\left(T_{n}\left(F\right)\right)}^{*}T_{n}\left(G\right)T_{n}\left(\Lambda \right)T_{n}\left(G^{*}\right)T_{n}\left(F\right)\right\} & ∼\left\{{\left(T_{n}\left(F\right)\right)}^{*}T_{n}\left(G\Lambda G^{*}F\right)\right\}\\ & =\left\{T_{n}\left(F^{*}\right)T_{n}\left(G\Lambda G^{*}F\right)\right\}∼\left\{T_{n}\left(F^{*}G\Lambda G^{*}F\right)\right\}. \end{array}$$

If ω∈R and $y\in C^{N\times 1}$, then,

$$y^{*}{\left(F\left(\omega \right)\right)}^{*}F\left(\omega \right)y={\left(F(\omega )y\right)}^{*}F\left(\omega \right)y={∥F\left(\omega \right)y∥}_{2}>0$$

whenever $F\left(\omega \right)y\neq 0_{N\times 1}$. As det(F(ω))≠0, $F\left(\omega \right)y=0_{N\times 1}$ if and only if $y={\left(F\left(\omega \right)\right)}^{-1}0_{N\times 1}=0_{N\times 1}$, hence ${\left(F\left(\omega \right)\right)}^{*}F\left(\omega \right)$ is positive definite for all ω∈R, and applying ([11], Theorem 4.4) and ([18], Corollary VI.1.6), yields:

$$0<\min_{\omega \in [0,2\pi ]}\lambda_{N}\left({\left(F\left(\omega \right)\right)}^{*}F\left(\omega \right)\right)=\underset{\omega \in [0,2\pi ]}{\inf}\lambda_{N}\left({\left(F\left(\omega \right)\right)}^{*}F\left(\omega \right)\right)\leq \lambda_{nN}\left(T_{n}\left(F^{*}F\right)\right)\;\forall n\in N.$$

Thus,

$$\begin{array}{cc} ∥{\left(T_{n}\left(F^{*}F\right)\right)}^{-1}{∥}_{2} & =\max_{k\in \{1,\cdots ,nN\}}\left|\lambda_{k}\left({\left(T_{n}\left(F^{*}F\right)\right)}^{-1}\right)\right|=\max_{k\in \{1,\cdots ,nN\}}\left|\frac{1}{\lambda_{k}\left(T_{n}\left(F^{*}F\right)\right)}\right|\\ & =\max_{k\in \{1,\cdots ,nN\}}\frac{1}{\lambda_{k}\left(T_{n}\left(F^{*}F\right)\right)}=\frac{1}{\lambda_{nN}\left(T_{n}\left(F^{*}F\right)\right)}\leq \frac{1}{\min_{\omega \in [0,2\pi ]}\lambda_{N}\left({\left(F\left(\omega \right)\right)}^{*}F\left(\omega \right)\right)} \end{array}$$

for all n∈N. Observe that $\left\{∥{\left({\left(T_{n}\left(F\right)\right)}^{*}T_{n}\left(F\right)\right)}^{-1}{∥}_{2}\right\}$ is also bounded, because:

$$\begin{array}{cc} ∥{\left({\left(T_{n}\left(F\right)\right)}^{*}T_{n}\left(F\right)\right)}^{-1}{∥}_{2} & =∥{\left(T_{n}\left(F\right)\right)}^{-1}{\left({\left(T_{n}\left(F\right)\right)}^{*}\right)}^{-1}{∥}_{2}={∥{\left(T_{n}\left(F\right)\right)}^{-1}{\left({\left(T_{n}\left(F\right)\right)}^{-1}\right)}^{*}∥}_{2}\\ & \leq ∥{\left(T_{n}\left(F\right)\right)}^{-1}{∥}_{2}∥{\left({\left(T_{n}\left(F\right)\right)}^{-1}\right)}^{*}{∥}_{2}={∥{\left(T_{n}\left(F\right)\right)}^{-1}∥}_{2}^{2}\;\forall n\in N. \end{array}$$

Moreover, from ([10], Theorem 3), we obtain $\left\{{\left(T_{n}\left(F\right)\right)}^{*}T_{n}\left(F\right)\right\}=\left\{T_{n}\left(F^{*}\right)T_{n}\left(F\right)\right\}∼\left\{T_{n}\left(F^{*}F\right)\right\}$. Consequently, applying Lemma A1 (see Appendix E) and ([11], Theorem 6.4), yields:

(A7) $$\left\{{\left({\left(T_{n}\left(F\right)\right)}^{*}T_{n}\left(F\right)\right)}^{-1}\right\}∼\left\{{\left(T_{n}\left(F^{*}F\right)\right)}^{-1}\right\}∼\left\{T_{n}\left({\left(F^{*}F\right)}^{-1}\right)\right\}=\left\{T_{n}\left(F^{-1}{\left(F^{*}\right)}^{-1}\right)\right\}.$$

Therefore, from Equation (A6), ([10], Lemma 2), and ([10], Theorem 3), we have:

$$\begin{array}{cc} \left\{{\left({\left(T_{n}\left(F\right)\right)}^{*}T_{n}\left(F\right)\right)}^{-1}{\left(T_{n}\left(F\right)\right)}^{*}T_{n}\left(G\right)T_{n}\left(\Lambda \right)T_{n}\left(G^{*}\right)T_{n}\left(F\right)\right\} & ∼\left\{T_{n}\left(F^{-1}{\left(F^{*}\right)}^{-1}\right)T_{n}\left(F^{*}G\Lambda G^{*}F\right))\right\}\\ & ∼\left\{T_{n}\left(F^{-1}G\Lambda G^{*}F\right))\right\}. \end{array}$$

Hence, applying Equation (A7), ([10], Lemma 2), and ([10], Theorem 3), we deduce that:

$$\begin{array}{cc} \left\{E\left(x_{n:1}x_{n:1}^{*}\right)\right\} & ∼\left\{T_{n}\left(F^{-1}G\Lambda G^{*}F\right))T_{n}\left(F^{-1}{\left(F^{*}\right)}^{-1}\right)\right\}\\ & ∼\left\{T_{n}\left(F^{-1}G\Lambda G^{*}{\left(F^{*}\right)}^{-1}\right)\right\}=\left\{T_{n}\left(F^{-1}G\Lambda G^{*}{\left(F^{-1}\right)}^{*}\right)\right\}=\left\{T_{n}\left(X\right)\right\}. \end{array}$$

(2) First, we prove that X(ω) is positive definite for all ω∈R. Fix ω∈R, and consider $y\in C^{N\times 1}$. Since $G\left(\omega \right)\Lambda {\left(G\left(\omega \right)\right)}^{*}$ is positive definite (see the proof of Theorem 2), we have:

$$\begin{array}{cc} y^{*}X\left(\omega \right)y & =y^{*}{\left(F\left(\omega \right)\right)}^{-1}G\left(\omega \right)\Lambda {\left(G\left(\omega \right)\right)}^{*}{\left({\left(F\left(\omega \right)\right)}^{-1}\right)}^{*}y\\ & ={\left({\left({\left(F\left(\omega \right)\right)}^{-1}\right)}^{*}y\right)}^{*}G\left(\omega \right)\Lambda {\left(G\left(\omega \right)\right)}^{*}{\left({\left(F\left(\omega \right)\right)}^{-1}\right)}^{*}y>0 \end{array}$$

whenever ${\left({\left(F\left(\omega \right)\right)}^{*}\right)}^{-1}y={\left({\left(F\left(\omega \right)\right)}^{-1}\right)}^{*}y\neq 0_{N\times 1}$. As ${\left({\left(F\left(\omega \right)\right)}^{*}\right)}^{-1}y=0_{N\times 1}$ if and only if $y={\left(F\left(\omega \right)\right)}^{*}0_{N\times 1}=0_{N\times 1}$, X(ω) is positive definite. Secondly, we prove that $E\left(x_{n:1}x_{n:1}^{⊤}\right)$ is positive definite for all n∈N, or equivalently, $\det\left(E\left(x_{n:1}x_{n:1}^{⊤}\right)\right)\neq 0$ for all n∈N. Applying Equation (A5) yields:

$$\begin{array}{cc} \det\left(E\left(x_{n:1}x_{n:1}^{⊤}\right)\right) & =\det({\left(T_{n}\left(F\right)\right)}^{-1}T_{n}\left(G\right)T_{n}\left(\Lambda \right)T_{n}\left(G^{*}\right){\left({\left(T_{n}\left(F\right)\right)}^{*}\right)}^{-1})\\ & =\frac{\det(T_{n}\left(G\right)T_{n}\left(\Lambda \right)T_{n}\left(G^{*}\right))}{\det\left(T_{n}\left(F\right)\right)\det\left({\left(T_{n}\left(F\right)\right)}^{*}\right)}=\frac{{\left(\det(\Lambda )\right)}^{n}}{|\det\left(T_{n}\left(F\right)\right){|}^{2}}={\left(\det(\Lambda )\right)}^{n}\neq 0\;\forall n\in N. \end{array}$$

The result now follows from Theorem 1. ☐

## Appendix E. A Property of Asymptotically Equivalent Sequences of Invertible Matrices

**Lemma** **A1.**
*Let $A_{n}$ and $B_{n}$ be nN×nN invertible matrices for all n∈N. Suppose that $\left\{A_{n}\right\}∼\left\{B_{n}\right\}$ and $\left\{∥A_{n}^{-1}{∥}_{2}\right\}$ and $\left\{∥B_{n}^{-1}{∥}_{2}\right\}$ are bounded. Then, $\left\{A_{n}^{-1}\right\}∼\left\{B_{n}^{-1}\right\}$.*

**Proof.** If M∈[0,∞) such that $∥A_{n}^{-1}{∥}_{2},{∥B_{n}^{-1}∥}_{2}\leq M$ for all n∈N, then:

$$\begin{array}{cc} 0 & \leq \frac{{∥A_{n}^{-1}-B_{n}^{-1}∥}_{F}}{\sqrt{n}}=\frac{{∥B_{n}^{-1}-A_{n}^{-1}∥}_{F}}{\sqrt{n}}=\frac{{∥B_{n}^{-1}A_{n}A_{n}^{-1}-B_{n}^{-1}B_{n}A_{n}^{-1}∥}_{F}}{\sqrt{n}}=\frac{{∥B_{n}^{-1}\left(A_{n}-B_{n}\right)A_{n}^{-1}∥}_{F}}{\sqrt{n}}\\ & \leq {∥B_{n}^{-1}∥}_{2}\frac{{∥\left(A_{n}-B_{n}\right)A_{n}^{-1}∥}_{F}}{\sqrt{n}}\leq {∥B_{n}^{-1}∥}_{2}\frac{{∥A_{n}-B_{n}∥}_{F}}{\sqrt{n}}{∥A_{n}^{-1}∥}_{2}\leq M^{2}\frac{{∥A_{n}-B_{n}∥}_{F}}{\sqrt{n}}\to 0. \end{array}$$

☐

This result was presented in ([6], Theorem 1) for the case N=1.
